# The impact of task difficulty on the lateralization of processing in the human auditory cortex

**DOI:** 10.1002/hbm.24776

**Published:** 2019-08-28

**Authors:** André Brechmann, Nicole Angenstein

**Affiliations:** ^1^ Special Lab Non‐Invasive Brain Imaging Leibniz Institute for Neurobiology Magdeburg Germany

**Keywords:** audition, categorization, contralateral noise procedure, hemispheric specialization, sequential processing, working memory

## Abstract

Perception of complex auditory stimuli like speech requires the simultaneous processing of different fundamental acoustic parameters. The contribution of left and right auditory cortex (AC) in the processing of these parameters differs. In addition, activity within the AC can vary positively or negatively with task performance depending on the type of task. This might affect the allocation of processing to the left and right AC. Here we studied with functional magnetic resonance imaging the impact of task difficulty on the degree of involvement of the left and right AC in two tasks that have previously been shown to differ in hemispheric involvement: categorization and sequential comparison of the direction of frequency modulations (FM). Task difficulty was manipulated by changing the speed of modulation and by that the frequency range covered by the FM. To study the impact of task‐difficulty despite covarying the stimulus parameters, we utilized the contralateral noise procedure that allows comparing AC activation unconfounded by bottom‐up driven activity. The easiest conditions confirmed the known right AC involvement during the categorization task and the left AC involvement during the comparison task. The involvement of the right AC increased with increasing task difficulty for both tasks presumably due to the common task component of categorizing FM direction. The involvement of left AC varied with task difficulty depending on the task. Thus, task difficulty has a strong impact on lateralized processing in AC. This connection must be taken into account when interpreting future results on lateralized processing in the AC.

**Abbreviations**
AC: auditory cortex
BA: Brodmann area
BOLD: blood‐oxygen‐level‐dependent
dGCM: directed Granger causality maps
EPI: echo planar imaging
FM: frequency modulation/modulated
fMRI: functional magnetic resonance imaging
GLM: general linear model
IFG: inferior frontal gyrus
MTG: middle temporal gyrus
RFX: random effects
ROI: region‐of‐interest
STG: superior temporal gyrus

## 1 INTRODUCTION

The perception of complex acoustic stimuli (e.g., speech, music) requires the simultaneous processing of different fundamental acoustic parameters such as frequency, duration, and intensity. Hemispheric specialization is presumably one way how the brain copes with this challenge of parallel processing in the auditory domain. Lateralization of processing in the auditory cortex (AC) does not only depend on the presented stimulus (bottom‐up effect) (e.g., Zatorre, Belin, & Penhune, 2002) but also on the given task (top‐down effect) (Angenstein & Brechmann, 2013a; Brechmann & Scheich, 2005; Scheich, Brechmann, Brosch, Budinger, & Ohl, 2007; Zatorre & Gandour, 2008). However, it is not fully understood how the lateralization of processing in AC varies with the difficulty of a given task. Most studies that varied task difficulty did not explicitly address changes of lateralization of processing due to this variation (Binder, Liebenthal, Possing, Medler, & Ward, 2004; Harinen & Rinne, 2013; Rinne, Koistinen, Salonen, & Alho, 2009; Rinne, Koistinen, Talja, Wikman, & Salonen, 2012) except for Reiterer et al. who directly compared parametric effects between the left and right hemisphere (Reiterer et al., 2005; Reiterer, Erb, Grodd, & Wildgruber, 2008). This variation is conceivable and many studies have shown that activity within the AC can vary positively or negatively with performance, depending on the type of task. In the following paragraph, we summarize such findings that directly relate to the AC in humans.

Activity in the right planum temporale inversely correlated with the performance of the participants during categorization of frequency modulation (FM) direction, that is, activity was lowest in participants who were most proficient in the task or in one participant who became more proficient with repeated measurements (Brechmann & Scheich, 2005). This inverse correlation was interpreted as a restriction to neurons specialized for the discrimination of FM direction at the expense of less specific neurons during high performance. In contrast, activity in the left planum temporale positively correlated with the performance during a two‐back working memory task regarding FM direction and pitch (Brechmann et al., 2007). This positive correlation was interpreted as a correlate of working memory possibly reflecting the maintenance of previous information by neurons that show sustained activity. Similarly, Gaab, Gaser, Zaehle, Jäncke, and Schlaug (2003) observed a positive correlation between accuracy in a pitch memory task and activity in the left and right supramarginal gyrus. A decrease of activity with increasing memory load for pitch has been observed by Rinne et al. (2009) in Heschl's gyrus and superior temporal gyrus (STG). Here, the hit rates decreased with increasing task difficulty while the reaction time decreased. The authors suggested that this was caused by an interruption of the pitch processing in order to save resources for the memory task. However, the correlations between activity and memory load do not seem to be specific for the pitch task, as they were also found in an auditory spatial memory task (Rinne et al., 2012) and in a vowel memory task (Harinen & Rinne, 2013). Reiterer et al. (2005) found an increase in activity with increasing accuracy among others regions in the right STG during comparisons of duration or pitch with varying difficulty within pairs of tones while the activity in the temporal lobe during both tasks was left lateralized. For intensity and timbre comparison with varying difficulty, they did not observe any significant correlation between activity and behavior in the cortex (Reiterer et al., 2008). During a frequency discrimination task, Holcomb et al. (1998) found in the left and right AC increasing regional cerebral blood flow when the participants became faster during the experiment. During a syllable detection task, Binder et al. (2004) observed in the left and right AC increasing activity with increasing signal‐to‐noise ratio (SNR) and increasing performance.

In summary, the activity in the AC seems to increase with increasing performance in tasks explicitly requiring auditory working memory (Brechmann et al., 2007; Gaab et al., 2003; Harinen & Rinne, 2013; Rinne et al., 2009; Rinne et al., 2012). However, the lateralization of this correlation is inconsistent. In discrimination tasks, both a decrease and an increase of AC activity with performance was observed (Binder et al., 2004; Brechmann & Scheich, 2005; Holcomb et al., 1998; Reiterer et al., 2005). It is therefore conceivable that a correlation between activity and performance is accompanied by a change in the lateralization of processing during changes in performance, for example, when the correlation is only present in one hemisphere, or when the correlations in the two hemispheres are opposite (one positive, one negative).

The aim of the present study was to determine the effect of task difficulty on the lateralization of processing in the human AC with functional magnetic resonance imaging (fMRI) during two tasks that differently involve the left and right AC, namely categorization and sequential comparison of FM direction. The categorization of tones based on their FM direction mainly involves the right AC (Behne, Scheich, & Brechmann, 2005; Brechmann & Scheich, 2005). The pairwise sequential comparison of FM direction requires the additional involvement of the left AC (Angenstein & Brechmann, 2013b), as well as stronger hemispheric interaction. The additional recruitment of the left AC in this task has been explained by working memory processes because a sound feature (i.e., FM‐direction) must be stored until the next tone is perceived and compared. This view is consistent with the special role of the left hemisphere in sequential processing documented in several studies (Bradshaw & Nettleton, 1981; Brechmann et al., 2007; Deike, Gaschler‐Markefski, Brechmann, & Scheich, 2004; Deike, Scheich, & Brechmann, 2010; Liegeois‐Chauvel, de Graaf, Laguitton, & Chauvel, 1999; Rosenthal, 2016).

In the present study, we varied the difficulty of these two tasks by changing the speed of modulation and by that, the frequency range of the FM. This way we changed the demand on identifying the direction of FM, which affects the difficulty of both the categorization and the comparison task. However, varying stimulus parameters to achieve different task difficulties introduces a confounding factor in determining the effects of task difficulty because of changes in activity elicited by stimulus‐dependent (bottom‐up) effects. Therefore, we utilized the contralateral noise procedure to determine the location of processing in the AC (Angenstein & Brechmann, 2013a; Angenstein & Brechmann, 2013b; Angenstein & Brechmann, 2015; Angenstein & Brechmann, 2017; Angenstein, Stadler, & Brechmann, 2016; Behne et al., 2005; Behne, Wendt, Scheich, & Brechmann, 2006; Stefanatos, Joe, Aguirre, Detre, & Wetmore, 2008). This procedure allows determining the involvement of left and right AC in task processing by using one set of stimuli in combination with only one task and without direct comparison of activity between hemispheres. The conventional direct comparison of activity between hemispheres, in contrast, is influenced by variations in the bottom‐up caused activity of different stimuli and also biased by anatomical differences between the left and right AC. The contralateral noise procedure exploits the contralaterality of the auditory pathway, that is, that the contralateral pathway dominates and suppresses the ipsilateral pathway (Brancucci et al., 2004; Kaneko, Fujiki, & Hari, 2003; Kimura, 1967). For this procedure, task‐relevant stimuli are presented without and with contralateral white noise. The addition of contralateral noise leads to a specific increase in activity in the AC that is involved in the given task when the task relevant stimuli are presented to the ipsilateral ear. This means, the location of this activity increase due to the additional contralateral noise uncovers the location of task processing.

We expected an involvement of the right AC during the categorization of FM direction and an involvement of the left and right AC during comparison of the FM direction when using task‐difficulties comparable to the previous studies (Angenstein & Brechmann, 2013b; Behne et al., 2005). For both tasks, we hypothesized an increase in the involvement of the right AC with increasing task difficulty based on the negative correlation between performance and activity in the study by Brechmann and Scheich (2005) because determining the FM direction is an integral initial part of both tasks. For the comparison task, we additionally hypothesized a decrease in the involvement of the left AC with increasing task difficulty based on the positive correlation of increasing activity in the left AC with increasing performance in the study by Brechmann et al. (2007). Note, however, that the working memory load was lower in the current study compared to the Brechmann et al. (2007) study that used a two‐back working memory task. In addition to the analysis with the contralateral noise procedure, we used binaural FM tone presentation as it is conventionally done within other studies that showed an effect of task difficulty in the AC. However, with this analysis we expect a stimulus‐dependent effect of the different frequency ranges of the tones for the different conditions of difficulty on the activity, which should lead to a decrease of activity with decreasing frequency range of the FM tones. Furthermore, we investigated how task difficulty affects connectivity from the left and right AC. We hypothesized that the connectivity from the left and right AC with the respective contralateral AC or other brain areas increased with its increasing involvement in the task.

## 2 MATERIALS AND METHODS

### 2.1 Participants

Sixteen right‐handed volunteers (Edinburgh Handedness Inventory; laterality quotient ≥40) with normal hearing (hearing level ≤ 15 dB from 125 to 6 kHz, interaural difference at each tested frequency ≤ 10 dB) were analyzed for the present study. Participants (age 20–34 years, mean age 28 years, seven females) gave written informed consent to the study, which was approved by the ethics committee of the University of Magdeburg. In all participants, speech processing was lateralized to the left hemisphere tested by an fMRI paradigm (Bethmann et al., 2007). Seven additional participants were excluded from the final analysis because their head movements during the fMRI‐measurement were stronger than 3 mm translation and/or 3° rotation or once more than 1 mm translation and/or 1° rotation from one volume to the next or more than 10 times 0.5 mm translation and/or 0.5° rotation from one volume to the next (two cases), their hit rates during the hardest conditions of the comparison task were below 70% (three cases), because of technical problems (two case).

### 2.2 Stimuli and task

Stimuli were 300 or 500 ms long, harmonic, linear frequency modulated tone complexes with five harmonics of decreasing amplitude (100% amplitude for fundamental frequency, 80% for second harmonic, 60% for third, 40% for fourth, 20% for fifth). The center frequencies (F_C_) of the fundamentals were 200, 240, 280 … 760 Hz. The frequency was either rising or falling. The starting and end frequency were calculated by F_C_*2^±(k * 0.5 * tone duration [s])^. Three different levels of difficulty were used depending on the speed of modulation: k = 1.5 (easy), k = 0.7 (medium) and k = 0.3 (hard). The stimuli were created with Matlab (The MathWorks Inc., Natick, MA) and CoolEdit 2000 (Syntrillium Software Corp., Phoenix, AZ).

During a single fMRI session, the FM tones were presented in 75 stimulation blocks of 15,800 ms each, which alternated with blocks of 12,200 ms silence. Within each stimulation block, 12 tones were presented with 1,000 ms of silence between the tones. Each block included six short tones and six long tones, and six upward FMs and six downward FMs. The tones within each block were presented either binaurally or monaurally to the right or left ear with or without continuous contralateral white noise. For each level of difficulty each of these five conditions were presented five times. The amplitude (root‐mean‐square, RMS) of the noise was 2 dB higher than the average amplitude of the monaurally presented tones. The amplitude of the binaurally presented FM tones was 4 dB lower than the amplitude of the monaurally presented tones in order to achieve a similar loudness percept. The five blocks for each of the 15 conditions (level of difficulty [3] × presentation mode [5]) were presented in pseudo‐randomized order such that two consecutive blocks never belonged to the same condition. Four different orders of presentation were randomized across participants.

The participants had to solve two tasks. Before the fMRI sessions in a psychoacoustic session with binaural presentation of FM tones, all participants were familiarized with the tasks and tested whether they could solve both tasks. The fMRI sessions with the two different tasks took place on two separate days. Some participants had to repeat sessions when their head movement exceed the defined quality criteria or technical problems occurred. In one fMRI session, they had to categorize the tones according to their direction of FM (upward vs. downward—categorization task). In another fMRI session, they had to compare the direction of FM between two consecutive tones and had to decide whether the direction changed from one to the next or was the same (comparison task). They had to press a button with their right index finger for one decision and another button with their right middle finger for the other decision. The stimuli/hand‐to‐button assignment was balanced across the group. The order of the two tasks was balanced across participants.

For stimulus presentation and recording of behavioral responses, the presentation software package (Neurobehavioral Systems, Albany) was used. The stimuli were presented via MRI compatible headphones (Baumgart et al., 1998). In addition, the participants wore earplugs. Before the experiments, the overall stimulus level was adjusted for each participant to a comfortable level and equally loud in both ears to avoid differences due to differences in the fitting of the earplugs. The participants decided by themselves at which level they could hear the stimuli clearly during the fMRI measurement.

### 2.3 Scanning procedure

The measurements were carried out on a 3 Tesla scanner (Philips Achieva dStream, Best, The Netherlands) equipped with a 32‐channel head coil. A 3D anatomical data set of the participant's brain (192 slices of 1 mm each, isotropic resolution) was obtained before the functional measurement. For fMRI 2,120 functional volumes were acquired in 35 min and 20 s using a continuous echo planar imaging (EPI) sequence (echo time [TE], 30 ms; repetition time [TR], 1,000 ms; flip angle, 60°; matrix size, 80 × 80; field of view, 24 × 24 cm^2^; 17 slices of 3 mm thickness with 0.3 mm gaps). The slices were oriented parallel to the Sylvian fissure that most of the temporal and parietal lobe was covered. The upper part of the frontal lobe, the upper part of the parietal lobe, the lower part of the occipital lobe and in very lowest part of the temporal lobe were not covered.

### 2.4 Data analysis

The functional data were analyzed using BrainVoyager™QX (Brain Innovation, Maastricht, Netherlands). A standard sequence of preprocessing steps, such as slice scan time correction, 3D‐motion correction, linear trend removal, and filtering with a high‐pass of two cycles per scan was performed. The functional data sets were co‐registered with the 3D‐data set. The data were transformed to Talairach‐space and spatially smoothed with a Gaussian filter with 4 mm full width at half maximum.

#### 2.4.1 Contralateral noise procedure

Random‐effects (RFX) analyses with a general linear model (GLM), including z‐transformed functional data of all 16 participants, were performed using the 2‐gamma response function as implemented in BrainVoyager™QX. Correction for serial correlation was performed using the second order autoregressive model (Goebel, 2012). For further analysis, a mask was created that exclusively included voxels with a significant positive change in the blood‐oxygen‐level‐dependent (BOLD) effect during stimulation (q[FDR] < 0.05; t ≥ 2.53). This procedure was chosen to ensure that only regions with a significant increase in BOLD signal during stimulation compared to silence periods were included in the further GLM analysis. To identify regions that were especially involved in the processing of the tasks at a specific level of difficulty, we searched for an increase in activity due to additional contralateral noise. The location of the activity increase due to contralateral noise during ipsilateral presentation of the task‐relevant stimuli reveals the location of task processing (Angenstein et al., 2016; Angenstein & Brechmann, 2013a; Angenstein & Brechmann, 2013b; Angenstein & Brechmann, 2015; Angenstein & Brechmann, 2017; Behne et al., 2005; Behne et al., 2006). For each difficulty level in each task two contrasts in BOLD signal were computed in order to show the increase in the BOLD signal due to the additional contralateral noise (t ≥ 3, cluster threshold: 150 mm^3^):

1. Left FM tones with noise > left FM tones without noise.
2. Right FM tones with noise > right FM tones without noise.

#### 2.4.2 ANOVA of the conditions with binaural tone presentation

The conditions with binaural presentation of tones were used to compute an analysis of variance (ANOVA) with the factors level of difficulty (easy, medium, and hard) and task (categorization vs. comparison) (t ≥ 8 corresponding to q[FDR] < 0.05 for the factor difficulty, cluster threshold: 150 mm^3^). RFX region‐of‐interest (ROI) GLM were computed for the resulting clusters in the AC and for the factor difficulty for all resulting cortical clusters in order to further characterize the effects.

#### 2.4.3 Connectivity analysis

For the connectivity analyses, the unmasked data were used and two seed regions were defined: one in the left and one in the right AC. A conjunction analysis was computed with the assumption that activity during all binaural tone presentation conditions was higher than during the silence periods. We defined two clusters of equal size (270 mm^3^), one in the left and one in the right AC including the most significant voxels (left: t ≥ 9; right: t ≥ 7.5; without interpolation). The clusters are located in the left hemisphere around Heschl's sulcus (Talairach coordinates, center of gravity: −46, −21, 6) and in the right AC on Heschl's gyrus (44, −19, 5). These ROIs were used as seeds for RFX Granger causality mapping (version 2.5, plugin in BrainVoyager QX) (Roebroeck et al., 2005). The directed Granger causality maps (dGCM) were computed for binaural tone presentation for each task and each level of difficulty. The resulting maps were compared between different levels of difficulty within one task with t‐tests (t ≥ 3; cluster threshold: 100mm^3^; without interpolation).

## 3 RESULTS

### 3.1 Behavior

Hit rates and reaction times (Table 1) of the conditions with monaural tones were subjected to analyses of variance (ANOVAs) with the factors side of presentation (left vs. right), noise condition (without vs. with contralateral noise), difficulty (easy, medium, and hard), and task (categorization vs. comparison) and post hoc two‐sided *t*‐tests were performed.

**Table 1:** Behavioral data of the 16 participants with *SE*

	Bilateral tones	Left tones	Right tones	Left tones with right noise	Right tones with left noise
Hit rate (%) categorization					
Easy level	95.6 ± 1.4	96.4 ± 1.0	97.0 ± 0.8	96.1 ± 1.1	95.4 ± 1.4
Medium level	95.0 ± 1.2	94.6 ± 1.1	95.8 ± 1.3	95.3 ± 1.5	95.5 ± 1.2
Hard level	91.6 ± 1.9	91.6 ± 1.6	92.4 ± 1.8	92.1 ± 1.4	91.1 ± 1.5
Hit rate (%) comparison					
Easy level	93.9 ± 1.2	95.3 ± 1.5	96.6 ± 0.8	96.0 ± 0.9	95.6 ± 1.4
Medium level	92.4 ± 1.5	92.0 ± 1.4	92.4 ± 1.9	91.1 ± 1.5	92.3.7 ± 1.9
Hard level	87.7 ± 2.1	85.3 ± 2.5	86.6 ± 2.5	84.1 ± 2.8	85.8 ± 2.3
Reaction time (ms) categorization					
Easy level	595.1 ± 15.8	594.0 ± 19.2	592.8 ± 16.3	583.3 ± 17.5	587.5 ± 14.2
Medium level	603.7 ± 17.0	611.7 ± 17.4	611.5 ± 15.9	601.5 ± 18.8	609.9 ± 17.6
Hard level	638.3 ± 17.0	639.9 ± 16.9	646.5 ± 15.9	626.0 ± 17.0	632.6 ± 12.1
Reaction time (ms) comparison					
Easy level	663.8 ± 15.1	657.6 ± 14.4	664.6 ± 15.8	644.9 ± 15.8	650.2 ± 17.9
Medium level	669.5 ± 13.7	684.2 ± 16.3	671.2 ± 16.4	661.1 ± 15.9	671.3 ± 16.7
Hard level	700.3 ± 14.9	699.0 ± 15.3	708.7 ± 18.4	699.3 ± 14.4	691.6 ± 15.2

The ANOVA of the hit rates revealed a significant main effect of task (F [1, 15] = 9.8; *p* = .007) with slightly higher hit rates during categorization (94.4 ± 1.0%) than during comparison (91.1 ± 1.4%), a significant main effect of difficulty (F [2, 14] = 17.6; *p* < .001) with lower hit rates for the more difficult conditions (easy: 96.1 ± 0.8; medium: 93.6 ± 1.2; hard: 88.6 ± 1.6; *p* < .001), and a significant interaction between task and difficulty (F [2, 14] = 12.4; *p* = .001). The hit rates differed strongest for the hardest conditions (*p* = .0008), slightly for the medium difficulty (*p* = .012) and did not differ for the easiest conditions between the tasks (*p* = .69).

The ANOVA of the reaction times revealed a significant main effect of task (F [1, 15] = 19.8, *p* < .001), difficulty (F [2, 14] = 62.7, *p* < .001) and noise (F [1, 15] = 13.3, *p* = .002), and a significant interaction between side of presentation, noise and difficulty (F [2, 14] = 4.0, *p* = .04). The reaction times were faster during categorization (611.4 ± 15.9 ms) than during comparison (675.3 ± 15.1 ms), and faster during the conditions with noise (638. 3 ± 13.3 ms) than during the conditions without noise (648.5 ± 14.2 ms), and faster during the easy conditions (621.87 ± 13.9 ms) than during the medium conditions (640.3 ± 14.0 ms) and the slowest during the hard conditions (667.9 ± 13.5 ms). The significant interaction was caused by different effects of the noise for different levels of difficulty depending on the side of tone presentation. For presentation of tones to the left ear, the reaction times were faster in the conditions with noise than without noise for the easiest (*p* = .02) and medium level of difficulty (*p* = .01). For presentation of tones to the right ear, the reaction times were faster in the conditions with noise than without noise for the hardest conditions (*p* < .01).

### 3.2 fMRI: Effect of task difficulty and task on the involvement of left and right AC revealed by the contralateral noise procedure

**Figure 1:**
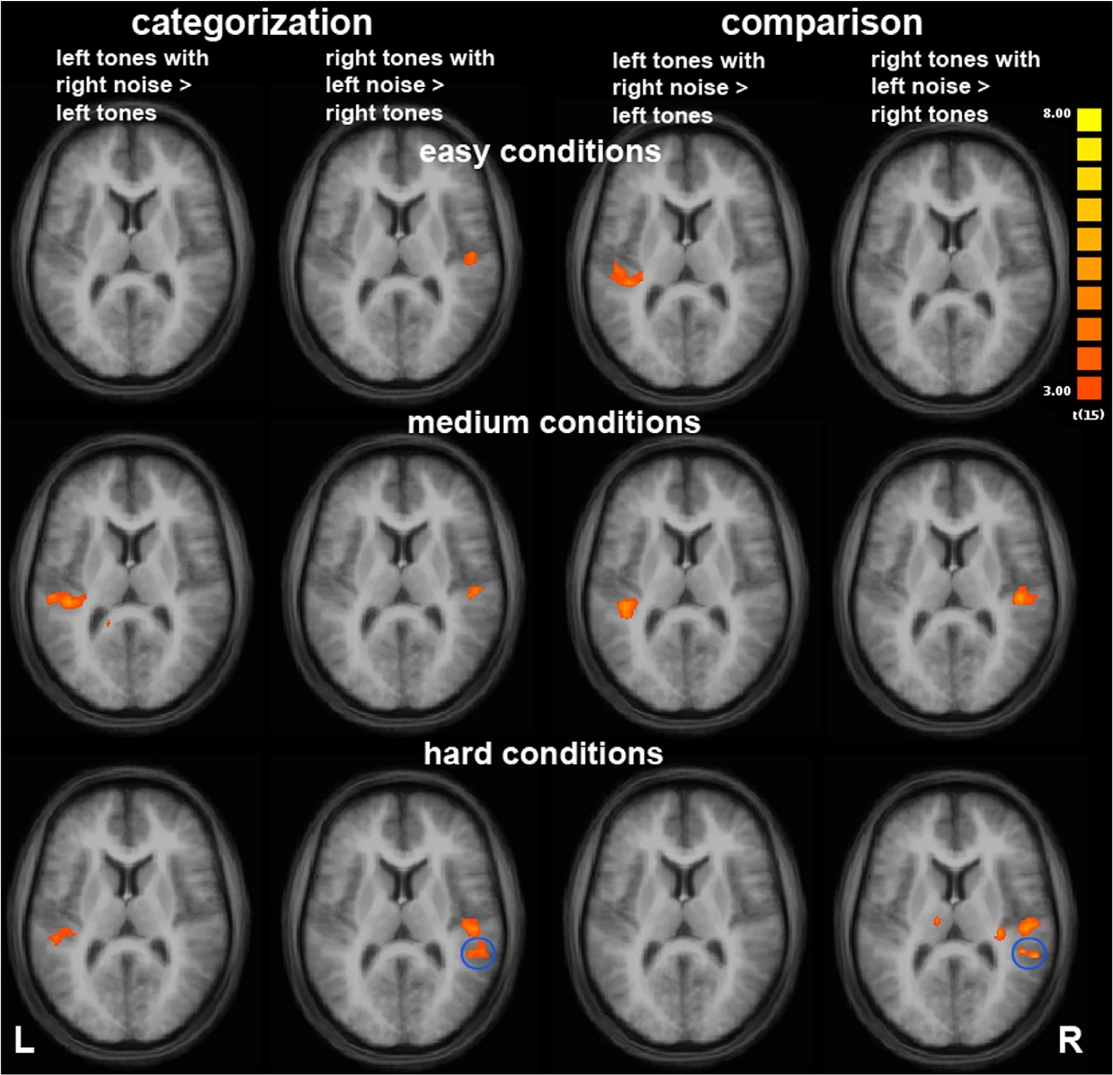
Activity increase due to additional presentation of contralateral noise during categorization or sequential comparison of tones based on their FM direction during presentation of FM tones to left and right ear (Talairach coordinate z = 10; t ≥ 3, cluster threshold: 150 mm^3^, *N* = 16). The activity increase varied with the level of difficulty depending on the task. The blue circles mark a separate cluster of activity in the posterior superior temporal gyrus that became significantly affected by noise at the highest level of difficulty in both tasks. FM, frequency modulation [Color figure can be viewed at http://wileyonlinelibrary.com]

The GLM revealed that the additional contralateral noise differently increased the activated volume during ipsilateral tone presentation in the left and right AC depending on the task and the level of difficulty (Figure 1, Table 2). This increase in activity due to noise points to the location of task processing (see Introduction). In the easy conditions, the noise affected the activity only in the right AC in the categorization task and only in the left AC in the comparison task. In the medium difficult conditions, the noise affected the activity of both the left and right AC during both tasks. For the hard conditions, the noise affected the activity in both ACs in the categorization task but only the activity in the right AC in the comparison task.

**Table 2:** Peak points within regions with an increase of activity due to additional contralateral noise of the group analysis with 16 participants (t ≥ 3, cluster threshold: 150mm³)

Location	BA	x	y	z	t	Volume [mm^3^]
**Categorization**		**Left tones with noise > left tones**				
**Easy level of difficulty**						
L midbrain		−6	−34	−9	10.5	802
Thalamus		−3	−10	4	5.0	210
R pons		9	−19	−20	4.7	379
**Medium level of difficulty**						
**L STG**	**42**	**−42**	**−28**	**10**	**5.8**	**1,651**
L cingulate gyrus	31	−21	−42	34	4.5	310
L posterior cingulate gyrus	29	−14	−43	13	3.9	207
L parahippocampal gyrus	30/27	−6	−34	−5	5.7	2,102
R precuneus	31	18	−49	31	4.6	180
**Hard level of difficulty**						
**L STG**	**22**	**−48**	**−31**	**7**	**5.2**	**877**
**L STG** (base of Heschl's gyrus)	**41/42**	**−36**	**−34**	**16**	**3.7**	**158**
**Categorization**		**Right tones with noise > right tones**				
**Easy level of difficulty**						
R midbrain		3	−37	−5	5.9	314
**R STG**	**22**	**48**	**−16**	**10**	**4.2**	**239**
**Medium level of difficulty**						
Midbrain		0	−37	−5	6.1	628
**R transverse temporal gyrus**	**42**	**48**	**−22**	**10**	**5.2**	**439**
**Hard level of difficulty**						
R midbrain		3	−34	−5	7.0	1,135
**R transverse temporal gyrus**	**42**	**48**	**−22**	**10**	**5.0**	**733**
**R STG**	**42/22**	**48**	**−40**	**16**	**4.3**	**717**
**Comparison**		**Left tones with noise > left tones**				
**Easy level of difficulty**						
**L STG**	**42/22**	**−42**	**−28**	**4**	**5.3**	**2,186**
L midbrain		−6	−35	−8	7.5	654
**Medium level of difficulty**						
**L STG**	**42**	**−42**	**−31**	**10**	**5.5**	**1,474**
L midbrain		−6	−35	−8	6.3	495
**Hard level of difficulty**						
L midbrain		−6	−34	−8	5.4	221
**Comparison**		**Right tones with noise > right tones**				
**Easy level of difficulty**						
R midbrain		6	−34	−9	5.8	229
**Medium level of difficulty**						
R midbrain		6	−34	−9	9.1	690
**R STG**	**42**	**45**	**−25**	**10**	**5.7**	**1,069**
**Hard level of difficulty**						
Thalamus		−12	−16	13	4.7	171
R midbrain		6	−34	−8	7	817
**R STG** (base of Heschl's gyrus)	**41/42**	**27**	**−25**	**14**	**5.6**	**647**
**R STG**	**22**	**54**	**−40**	**10**	**5.8**	**793**
**R STG**	**42**	**48**	**−16**	**7**	**5.6**	**1,040**

*Note*: Areas of the auditory cortex are bold.

Abbreviations: BA, Brodmann area; x, y, z, Talairach coordinates; L, left; R, right; STG, superior temporal gyrus.

The effect of noise on activity in the right AC increased with increasing task difficulty for both tasks. In addition, during the hardest conditions, an additional area in the right planum temporale got significantly affected by the noise. During the comparison task, an additional area at the base of right Heschl's gyrus was significantly affected by the noise.

The effect of noise on the activity in the left AC during the categorization task was stronger for the medium difficult than for the most difficult conditions and absent for the easy conditions. During the comparison task, the effect of noise on the activity decreased with increasing task difficulty. During the categorization task, an additional area at the base of left Heschl's gyrus was significantly affected by the noise.

### 3.3 MRI: Effect of task difficulty and task on activity during binaural tone presentation

The ANOVA with the conditions with binaural tone presentation with the factors difficulty and task revealed significant effects for both factors in the left and right AC and further brain regions (Table 3).

**Table 3:** Peak points within regions that are significantly affected by the factors task or difficulty during binaural tone presentation of the group analysis with 16 participants revealed by the ANOVA (t ≥ 8, cluster threshold: 150mm^3^)

Location	BA	x	y	z	t	Volume [mm^3^]
**Difficulty** (t ≥ 8)						
L parietal operculum	40	−56	−19	19	13.9	576
**L STG**	**42**	**−42**	**−31**	**7**	**10.4**	**180**
L lentiform nucleus		−30	−18	1	27.2	1,030
Midbrain		0	−9	−14	15.2	206
R midbrain		3	−22	−5	13.4	271
R thalamus		21	−13	16	11.1	233
R claustrum		30	17	7	12.2	151
R middle frontal gyrus	6/9	42	5	25	11.3	178
**R STG**	**41/42**	**51**	**−22**	**16**	**14.0**	**693**
**Task** (t ≥ 8)						
**L STG (1)**	**42/22**	**−54**	**−25**	**7**	**22.4**	**794**
**L STG (2)**	**22**	**−54**	**−40**	**9**	**11.2**	**161**
L inferior parietal lobule	40	−48	−34	43	57.4	6,648
L precentral gyrus	6	−45	2	25	19.7	217
L claustrum		−27	26	1	23.7	934
L parahippocampal gyrus	28	−22	−22	−8	28.5	300
Thalamus+brainstem		−12	−25	10	58.0	15,577
L precuneus	7	−9	−73	52	21.8	1,724
R precuneus	7	6	−65	49	14.3	256
R parahippocampal gyrus	28	19	−28	−8	10.7	158
R anterior insula	13	30	20	1	18.9	529
R inferior parietal lobule	40	39	−40	40	33.1	3,454
R MTG	22	42	−31	−5	16.5	471
R IFG	44	54	5	19	17.0	425
**R STG (partly AC)**	**42**/22	**66**	**−22**	**13**	**33.4**	**3,199**

*Note*: Areas of the auditory cortex are bold.

Abbreviations: BA, Brodmann area; x, y, z, Talairach coordinates; L, left; R, right; IFG, inferior frontal gyrus; MTG, middle temporal gyrus; STG, superior temporal gyrus.

**Figure 2:**
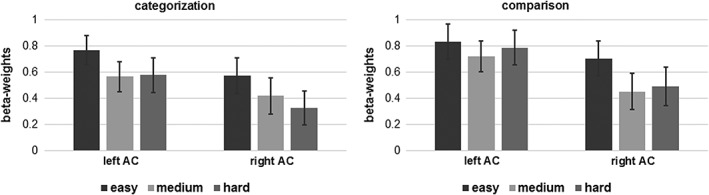
Beta‐weights with *SE* of the binaural FM tone presentation conditions of the regions within left and right AC that showed with the ANOVA an effect of task difficulty for the categorization and comparison task (t ≥ 8). AC, auditory cortex; ANOVA, analysis of variance; FM, frequency modulation

The ROI‐GLM for the clusters that showed an effect for the factor difficulty, within the AC regions revealed stronger activity for the easier conditions compared to both the medium (left AC: *p* = .00009; right AC: *p* = .001) and the hardest conditions (left AC: *p* = .009; right AC: *p* = .0003) (Figure 2). The left parietal operculum showed stronger activity for the easiest conditions compared to both the medium (*p* = .0007) and hardest conditions (*p* = .0001). In contrast, the right middle frontal gyrus showed the strongest activity for the hardest conditions compared to the medium (*p* < .02) and easiest conditions (*p* < .0005).

The ROI‐GLM within the AC regions that showed a main effect of task revealed stronger activity for the comparison than for the categorization task (left AC: ROI1: *p* = .008; ROI2: *p* = .01; right AC: *p* = .0005).

### 3.4 fMRI: Connectivity analysis

A comparison of the dGCMs of the binaural tone presentation conditions between different levels of difficulty revealed changes in connectivity from the left and right AC to ipsilateral and contralateral regions (Table 4). However, a change in connectivity between the left and right AC with increasing difficulty was not observed. During the categorization task, the connectivity from the right AC mainly increased interhemispherically to contralateral frontal and cingulate regions with increasing task difficulty. From the left AC, the connectivity mainly decreased intra‐ and interhemispherically to frontal and parietal regions with increasing task difficulty. For the comparison task, there were fewer differences in connectivity between the different levels of difficulty. Here, connectivity increased with increasing difficulty from the right AC to contralateral temporal region and cingulate gyrus.

**Table 4:** Regions of frontal, temporal, parietal cortex and cingulate gyrus with significant differences in dGCM between different levels of difficulty with seed located in the left or right AC (t ≥ 3, cluster threshold: 100mm^3^),

Location	BA	x	y	z	t	Volume (mm^3^)
Increasing connectivity with increasing task difficulty						
Categorization, seed in left AC, medium > easy						
R precuneus	7/31	2	−54	35	3.69	135
Categorization, seed in right AC, hard > medium						
L middle frontal gyrus	47	−47	39	−2	3.62	243
L cingulate gyrus	31	−17	−54	27	3.71	432
L medial frontal gyrus	10	−11	51	−6	3.98	135
Categorization, seed in right AC, hard > easy						
L cingulate gyrus	31	−13	−54	29	3.93	162
Comparison, seed in right AC, medium > easy						
L anterior cingulate	25	−2	13	−10	3.60	108
Comparison, seed in right AC, hard > easy						
L angular gyrus	39	−58	−57	29	3.09	108
L cingulate gyrus	24	−24	−18	37	4.15	189
Decreasing connectivity with increasing task difficulty						
Categorization, seed in left AC, medium > hard						
L MTG	21	−57	−48	−2	3.48	108
R precuneus	7	10	−45	56	3.80	108
R inferior parietal lobule	40	32	−39	47	3.77	189
Categorization, seed in left AC, easy > medium						
L IFG	45	−58	23	14	4.76	162
Categorization, seed in left AC, easy > hard						
L IFG	44	−50	12	6	3.36	108
R superior parietal lobe	7	35	−57	59	3.69	108
Categorization, seed in right AC, easy > medium						
R cingulate gyrus	31	19	−48	35	3.53	162

Abbreviations: BA, Brodmann area; x, y, z, Talairach coordinates; L, left; R, right; IFG, inferior frontal gyrus; MTG, middle temporal gyrus.

## 4 DISCUSSION

### 4.1 Effect of task difficulty on the involvement of the left and right AC revealed by the contralateral noise procedure

The results showed that the involvement of the left and right AC strongly depends on task difficulty for both categorization and comparison of FM direction. This was evidenced by the different effects of contralateral noise on the activity in the AC. The contralateral noise procedure reveals the involvement of the AC in a given task by reducing the signal‐to‐noise ratio during ipsilateral presentation of the task relevant stimuli (Angenstein et al., 2016; Angenstein & Brechmann, 2013a; Angenstein & Brechmann, 2013b; Angenstein & Brechmann, 2015; Angenstein & Brechmann, 2017; Behne et al., 2005; Behne et al., 2006). The longer reaction times and lower hit rates for the more difficult conditions indicates that the variation of difficulty by changing the modulation rate and by that the frequency range of the FM tone was successful.

In the easiest conditions, the contralateral noise procedure revealed an involvement of the right AC in categorization and an involvement of the left AC in sequential comparison of FM direction. In the medium difficult conditions, the left and right AC were significantly involved in both tasks. In the most difficult conditions, we found an involvement of the left and right AC in the categorization task and an involvement of the right AC in the comparison task. The results for the easiest conditions are partly in accordance with previous studies (Angenstein & Brechmann, 2013b; Behne et al., 2005) where the contralateral noise procedure revealed an involvement of the right AC in categorization of FM direction (Behne et al., 2005) and an involvement of the left and right AC in the comparison of FM direction (Angenstein & Brechmann, 2013b). The correspondence with the results of the easiest conditions of the present study was expected as we used the same speed of modulation for the FM tones like in the previous studies for the easiest conditions (Angenstein & Brechmann, 2013b; Behne et al., 2005). However, for the comparison task the present results suggest a main involvement of the left AC in contrast to the involvement of both AC in the previous study (Angenstein & Brechmann, 2013b). This difference may be due to differences in stimuli and/or task: The previous study included only one level of difficulty, the FM tones were 100 ms longer and presented in pairs so that pairwise comparison was required in contrast to the continuous 1‐back comparison in the present study, which may have increased the demand on working memory. Further studies focusing on fewer conditions are needed to test whether the lack of effect in the right AC is due to differences in stimuli and task or limited statistical power due to a limited number of participants.

In the right AC, the effect of noise increased with increasing difficulty in both tasks. This is accordance with our hypothesis that the involvement of the right AC in task processing increases with increasing task difficulty because categorization of FM direction is an integral part of both tasks and the right AC is specialized for it (Behne et al., 2005; Brechmann & Scheich, 2005). This increasing involvement of the right AC fits to the inverse correlation between activity in the right AC and performance shown in the study by Brechmann and Scheich (2005). We found an additional cluster within posterior STG that became involved in the processing of both tasks during the hardest conditions. The location of this cluster fits to the region on planum temporale that showed an inverse correlation between performance and activity in the previous study by Brechmann and Scheich (2005). This auditory association area was suggested to be specifically involved in the categorization of FM direction and it seems to be more important when the task gets harder. It possibly functions as interface between top‐down and bottom‐up processing to meet the demand of selective attention to a specific sound feature (FM‐direction). The categorization of the FM direction has to be performed in both tasks since it is also a prerequisite for the sequential comparison task. The categorization is most difficult when the frequency range is the smallest. This suggests that the categorization part drives the strong involvement of the right AC also for the comparison task in the most difficult condition.

In the left AC, the noise mainly had an effect on an area around Heschl's gyrus and spreading in some conditions posterior to the planum temporale. For the comparison task, the effect of noise in the left AC was the strongest for the easy conditions, then decreased to the medium conditions and was absent in the hardest conditions. This decrease of involvement of the left AC in the comparison task from the easy to the hard conditions is consistent to the positive correlation between activity in the left planum temporale and performance in the two‐back working memory task of the study by Brechmann et al. (2007). In the present study, the involvement of the left AC was strongest for the easiest conditions with the highest performance. The absence of a significant involvement of left AC during the hardest conditions suggests that the involvement of the right AC in categorization may be the determining factor for the distribution of processing between hemispheres in this situation. According to a hypothesis by Banich (1998), the division of labor between the hemispheres is always a balance between costs and benefits of hemispheric interaction. This hypothesis suggests that a division of labor between hemispheres can be beneficial when the processing demand is high and the capacity of one hemisphere is exceeded. This hypothesis is derived from visual studies. It has been shown that it also holds for auditory processing (Scalf, Banich, & Erickson, 2009). The cost of a balanced division of labor between the right and left AC during the comparison task may be too costly if the categorization of FM is very difficult and therefore the left AC involvement in the task is reduced. Whether this is due to the special listening situation where information about the FM direction is only available to the left AC after transfer from the right AC or via the weak ipsilateral pathway remains to be determined.

For the categorization task, there was no effect of the noise on activity in the left AC for the easy conditions as expected from previous studies (Behne et al., 2005; Brechmann & Scheich, 2005). However, when the task gets harder we found an involvement of the left AC although it is not specialized for the task. This may be explained by the theory of Banich (1998) that a division of labor between hemispheres can be beneficial when the processing demand is high and the capacity of one hemisphere is exceeded. However, the involvement of the left AC during categorization for the hardest conditions slightly decreased again. For the hardest conditions an effect on an additional region at base of Heschl's gyrus was observed (Table 2). The location of this area corresponds to an area in the right AC area that was additionally affected by the noise during the most difficult conditions of the comparison task. According to its location this area corresponds to the caudolateral (CL) and caudomedial (CM) areas defined in monkey AC (Kaas & Hackett, 2000). Hackett et al. (2014) suggest that CL and CM besides area Tpt may be the most dominant sources of inputs from the superior temporal cortex to posterior parietal areas. This may fit to the result that the connectivity from the AC to parietal areas changes with variation of task difficulty (see section 4.4).

In the present study, we observed a significant effect of the noise on the hit rates and reaction times. The participants seem to be better with contralateral noise, the hit rates increased and the reaction times decreased with noise. This was only observed once for the reaction times in a previous study (Angenstein & Brechmann, 2013a). This suggests that in the conditions with noise the lowered signal‐to‐noise ratio on a behavioral level seems to be overcompensated by the increase in activity in the ipsilateral AC. In previous studies, such a behavioral effect was mostly not significant (Angenstein et al., 2016; Angenstein & Brechmann, 2013b; Angenstein & Brechmann, 2015; Angenstein & Brechmann, 2017; Behne et al., 2005; Behne et al., 2006) except for (Angenstein & Brechmann, 2013a). The hypothesis is supported by the fact that the participants in the present study reported in half of the difficulty ratings that the conditions with noise were more demanding than the conditions without noise and nobody reported the reverse. This interpretation may also fit to the significant interaction between noise, difficulty, and side of presentation for the reaction times. Here the reaction times were faster with noise than without noise during the hardest condition when the FM tones were presented on the right ear and for both tasks the right (ipsilateral) AC was the strongest involved. The same effect of noise for the easy and medium condition during FM tone presentation to the left ear is not so easy to interpret because the lateralization of involvement of the left and right AC was mixed.

### 4.2 Effect of task difficulty on activity in the AC during binaural tone presentation

In this paragraph, we discuss the results of the effect of task difficulty when the tones are presented binaurally which conforms to the conventional approach for testing the functional organization of the AC. During binaural FM tone presentation, the activity within the AC was stronger for the easiest conditions compared to the more difficult conditions (Figure 2). Thus, the binaural FM tone conditions seems to mainly reveal the bottom‐up activity. The tones of the most difficult conditions with the slowest speed of FM have the smallest frequency range and hence cause the smallest bottom‐up activity. The tones of the easiest conditions with the fastest speed of FM have the largest frequency range and hence cause the largest bottom‐up activity. However, the activity between the most difficult and the medium difficult conditions did not differ. So either, the differences between the bottom‐up effects were weaker between these two conditions than between the easiest and the medium difficult conditions or the activity differences depended on an interaction of bottom‐up and top‐down effects. For the top‐down effect, we would expect increasing activity with increasing task difficulty. Together with the bottom‐up effect of decreasing activity with increasing task difficulty this could have led to the absence of activity differences between the medium and the most difficult conditions. This suggests that the binaural FM tone presentation leads to activity that is influenced by both bottom‐up effects (frequency range) and top‐down effects (task difficulty) and these effects are not separable.

As the change in the bottom‐up effect of frequency range cannot be avoided to increase the task difficulty, these results support the necessity to use the contralateral noise procedure for revealing involvement of the AC in the task. The use of the contralateral noise procedure reduces stimulus‐dependent effects as we do not compare conditions with different frequency ranges of the FM tones directly but rather compare conditions with the FM tones of the same frequency range (conditions without noise vs. conditions with contralateral noise).

Regions in the AC that showed an effect of task showed stronger activity for the comparison than for the categorization task which is consistent to previous findings (Angenstein & Brechmann, 2015; Angenstein & Brechmann, 2017). This also fits to the longer reaction times and lower hit rates during the comparison task suggesting that this task was more demanding than the categorization task. These differences in reaction time and hit rates also fits to the participant's experience that the comparison task was more difficult.

### 4.3 Effect of task difficulty on activity in cortical areas outside AC during binaural tone presentation

In the right middle frontal gyrus the strongest activity was observed for the most difficult conditions. So here, the binaural tone presentation conditions seems to mainly reveal a top‐down effect of task difficulty. The involvement of this region probably increases with increasing task difficulty because of an increasing demand on selective attention (Petersen & Posner, 2012) on the FM direction. This region is also discussed in studies on working memory (Rottschy et al., 2012). Here, the longer reaction time during the more difficult conditions is consistent with an increasing demand on working memory. An effect of task difficulty on the activity in the middle frontal gyrus was observed in two studies involving temporal tasks (Lewandowska, Piatkowska‐Janko, Bogorodzki, Wolak, & Szelag, 2010; Tregellas, Davalos, & Rojas, 2006). Lewandowska et al. (2010) found an increase in activity with increasing difficulty in an auditory temporal‐order‐judgment task besides other regions in the left middle frontal gyrus. Although we also found an effect of difficulty in this region, the location and the hemispheric distribution of this activity cluster was different. Lewandowska et al. (2010) suggested that areas with a decrease of activity with increasing task difficulty are more specific for timing whereas areas with an increase of activity with increasing task difficulty are related to nontemporal cognitive resources that are probably also required during our present tasks. In another study on temporal discrimination that involved sequential processing, also an increase in activity for the difficult compared to the easy condition was found in a similar region of the right dorsolateral prefrontal like in the present study besides a lot of other regions (Tregellas et al., 2006). In contrast, in this region such an effect of task difficulty was not found in other studies for example with pitch memory (Gaab et al., 2003; Reiterer et al., 2005; Rinne et al., 2009), frequency discrimination (Holcomb et al., 1998) or syllable detection (Binder et al., 2004).

In the left parietal operculum, the strongest activity was observed in the easiest conditions, with FM tones covering the largest frequency range. So here, the binaural tone presentation conditions seem to be dominated by the bottom‐up effect of task difficulty, that is, increasing activity with decreasing difficulty because of the increasing frequency range of the stimuli. From its location the area corresponds to parietal opercular areas OP 1 and OP 4 (Eickhoff, Amunts, Mohlberg, & Zilles, 2006). Especially the left OP 4 has been suggested to play an important role in the integration of functional coupling between primary AC and motor cortex presumably important for language processing (Sepulcre, 2015). The connection of OP 4 to the AC may be the reason that in the present study the activity in the parietal operculum showed the same pattern of activity as the AC, that is, stronger activity during the easier conditions. A meta‐analysis revealed that in studies using auditory stimulation activity in this area increased from nonexperts to experts (Neumann, Lotze, & Eickhoff, 2016). This may be comparable to the present situation that when the tasks get easier the activity increases. However, it is complicated to assign the region to OP 1 or OP 4 alone. This problem may be caused by the two closely neighboring activation sites that are often coactivated (Eickhoff et al., 2006). OP 1 is more likely connected to parietal regions and interhemispherically whereas OP 4 is more densely connected to frontal and primary sensory‐motor areas (Eickhoff et al., 2010). Therefore, Eickhoff et al. suggests that OP 1 is involved in more integrative aspects of somatosensory processing.

Consistent to previous studies, areas outside the AC also showed stronger activity during comparison than during categorization, for example, inferior parietal lobule, precentral gyrus, precuneus, anterior insula, and inferior frontal gyrus (Angenstein & Brechmann, 2015; Angenstein & Brechmann, 2017). As discussed above the longer reaction times and lower hit rates during the comparison task and the reports of the participants indicate that this task was more demanding than the categorization task. However, the larger differences of the hit rates between the tasks during the harder conditions are not reflected in activity differences. At the chosen level of significance, we did not observe areas with an interaction between task and difficulty.

### 4.4 Effect of task difficulty on connectivity

The connectivity of neither the left nor the right AC to the respective contralateral AC changed significantly when varying the difficulty of the two tasks. However, there are significant changes in connectivity between ACs and areas outside the AC. Overall, we found that right AC showed increasing connectivity with increasing task difficulty whereas connectivity from the left AC mainly decreases with increasing task difficulty (Table 4).

The connectivity increase with increasing task difficulty from the right AC mainly concerns regions of the default mode network, that is, anterior and posterior cingulate gyrus and precuneus (Raichle, 2015) as well as left middle frontal gyrus and left angular gyrus. Possible interpretations of the stronger connectivity from the right AC to these areas are the roles of the frontal and cingulate regions in attentional control (Leech & Sharp, 2014) and decision‐making (Padoa‐Schioppa & Conen, 2017), the role of the ventral division of the left angular gyrus in top‐down mechanisms across numerous tasks (Seghier, 2013), and the role of the left middle frontal gyrus in semantic processing (Binder, Desai, Graves, & Conant, 2009).

The connectivity decrease with increasing task difficulty from the left AC mainly concerns regions involved in semantic processing, that is, inferior frontal gyrus, inferior parietal lobe, posterior middle temporal gyrus (Bethmann et al., 2007; Binder et al., 2009). These regions may be involved in verbalizing the FM direction. Connectivity from the left AC also decreases with increasing task difficulty to contralateral parietal regions. Overall, this decrease in connectivity from the left AC with increasing task difficulty to other brain suggests that the effect of the left AC on other brain regions is downregulated with increasing task difficulty. As the right AC is specialized for categorization of FM direction the left AC may be prevented from disturbing the processing within the right AC by this decrease in connectivity. However, this interpretation is in contrast to the fact that the left AC gets involved in the categorization task when the task gets harder. Further studies with only one of the two tasks are needed to exclude potential transfer effects between the two different types of tasks and should test the present hypothetical interpretations. Finally, it cannot be ruled out that the bottom‐up effect on the activity in the AC had an influence on the results of the connectivity analysis.

## 5 CONCLUSION

The involvement of the AC in categorization and comparison of FM direction strongly changes with variations in task difficulty. This should be considered in studies investigating lateralized processing in the AC. The involvement of the right AC increases with increasing task difficulty for both tasks whereas the involvement of left AC varies with task difficulty depending on the task. In contrast to binaural FM tone presentation, the contralateral noise procedure enables the determination of the involvement of the left and right AC in task processing for different levels of difficulty relatively independent of the bottom‐up effect of the different frequency ranges for different level of difficulty. During binaural tone presentation, an effect of task difficulty unconfounded by bottom‐up effects can otherwise only be observed across participants with different levels of performance during presentation of the same stimuli or comparing activity between repeated practicing sessions.

## Data Availability

The data that support the findings of this study are available upon request from the Leibniz Institute for Neurobiology Magdeburg, Combinatorial NeuroImaging Core Facility (http://www.lin-magdeburg.de/cni) that organize institutional data access for external researchers.
